# Ileal gastrointestinal stromal tumor causing mesenteric gangrene

**DOI:** 10.1002/ccr3.5772

**Published:** 2022-04-20

**Authors:** Hazem Beji, Mahdi Bouassida, Slim Zribi, Hassen Touinsi

**Affiliations:** ^1^ Department of General Surgery Hospital Mohamed Taher Maamouri Nabeul Tunisia

**Keywords:** acute abdomen, gastrointestinal stromal tumor, infection, mesenteric gangrene

## Abstract

To our knowledge, this is one of the rare literature reports of an ileal GIST complicated with mesenteric gangrene. We reported successful surgical treatment. Infection of GIST is an extremely rare complication that should be treated with no delays to avoid rupture and peritonitis.

## Abstract 2

Ileal gastrointestinal stromal tumors are rare. Mesenteric gangrene is an extreme rare complication. Diagnosis is difficult. Surgical treatment should not be delayed.

## Graphical abstract

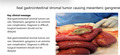

**Question:**What is shown in the intraoperative view?

**Answer**: Ileal gastrointestinal stromal tumor causing mesenteric gangrene.

A 45‐year‐old male patient was presented to the emergency department with acute abdominal pain. On examination, he had a temperature of 38.5°C. There was periumbilical tenderness. Laboratory studies showed a white blood count of 13800 cells/mm^3^ and the C reactive protein was raised to 65 mg/L. Computed tomography (CT) scan showed irregular wall thickening of the small intestine proximal to a hydroaeric mesenteric collection. We performed a laparotomy and found an ileal tumor (Figure 1) with gangrene in the contralateral mesenteric border (Figure 2). We performed ileal resection with end‐to‐end anastomosis.

**FIGURE 1:**
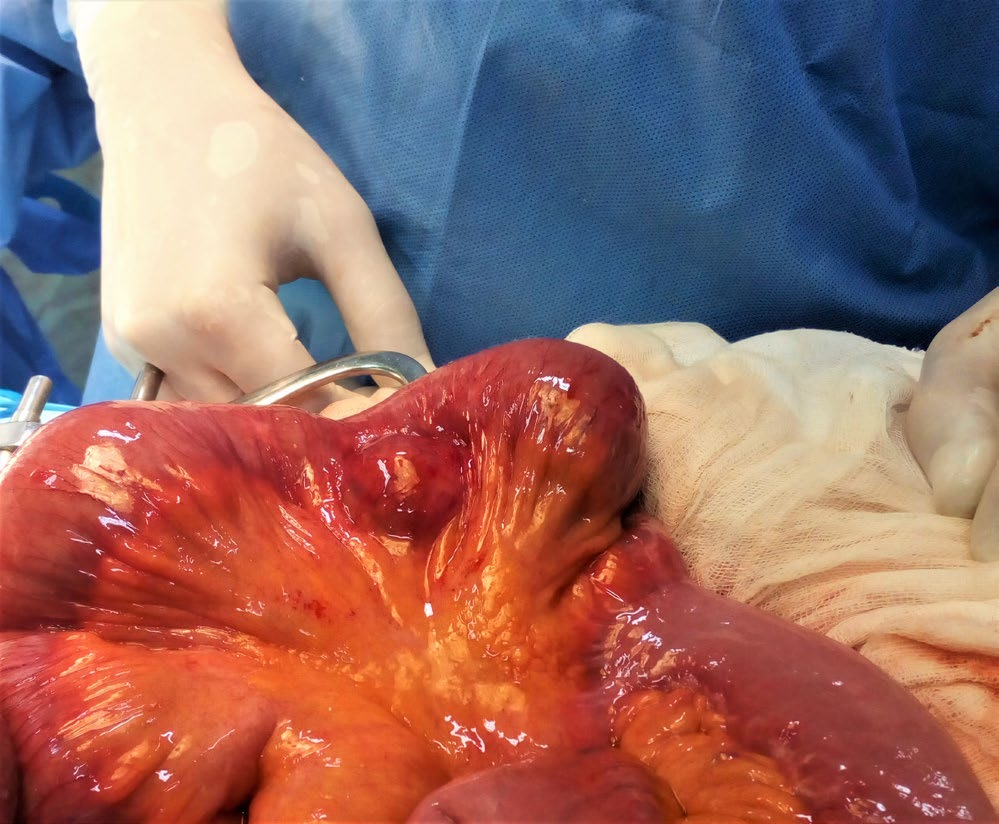
Intraoperative view showing the ileal tumor

**FIGURE 2:**
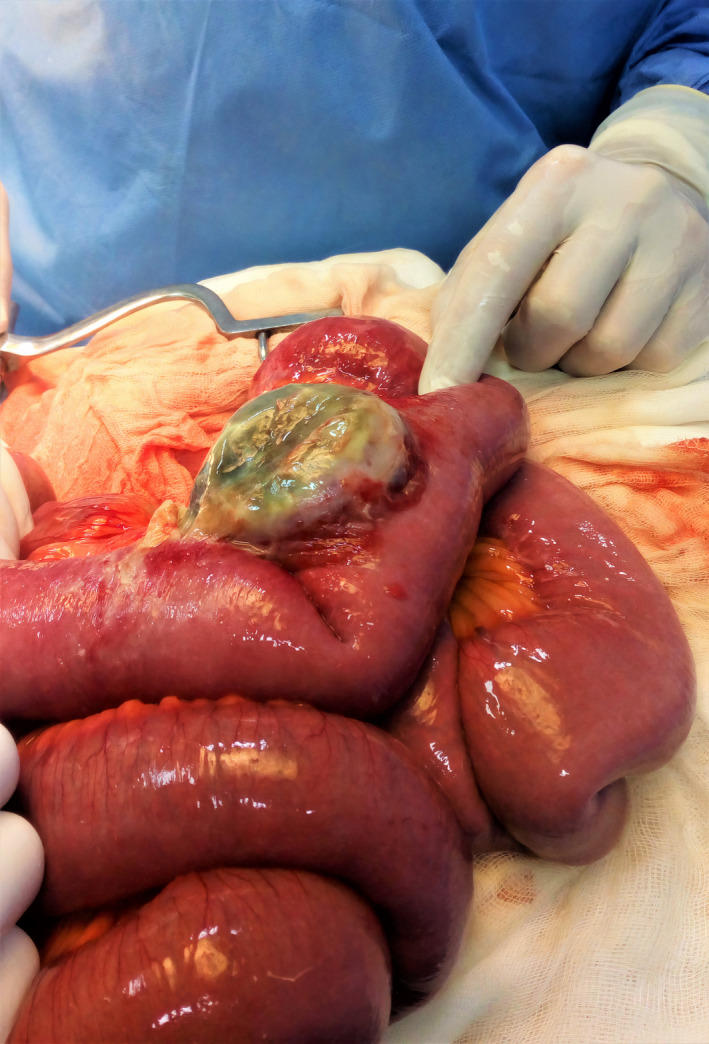
Intraoperative view showing mesenteric gangrene

The histopathologic examination showed spindle cells type GIST of the small intestine. There was a proliferation of cells with elongated nuclei and eosinophilic cytoplasm arranged in fascicles and expressing Dog1 in the immunohistochemistry (Figure 3). It was classified with a high risk of recurrence in Miettenen's classification.^1^ Resection margins were negative.

**FIGURE 3:**
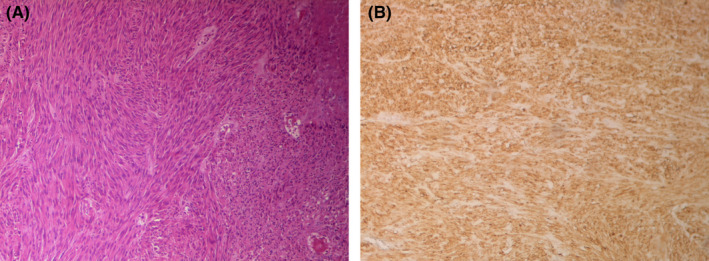
(A) Spindle cell type GIST of the small intestine: Proliferation of cells with elongated nuclei and eosinophilic cytoplasm arranged in fascicles (HEx40). (B) Cells expressing Dog1 in the immunohistochemistry (IHCX100)

Postoperatively, the patient was treated with Imatinib. He had an uneventful recovery with a follow‐up of 6 months. To the best of our knowledge, this is one of the rare literature reports of an ileal GIST complicated with mesenteric gangrene. We reported successful surgical treatment. Infection of GIST is an extremely rare complication that should be treated with no delays to avoid rupture and peritonitis.

## CONFLICT OF INTEREST

The authors declare that they have no conflict of interest.

## **AUTHOR** **CONTRIBUTIONS**

Hazem Beji and Mahdi Bouassida collected the data and involved in writing the manuscript. Slim Zribi edited the manuscript. Hassen Touinsi involved in validation.

## ETHICAL APPROVAL

This work meets ethical and legal guidelines.

## CONSENT

Written informed consent was obtained from the patient to publish this report in accordance with the journal's patient consent policy.

## Data Availability

Data sharing is not applicable to this article as no new data were created or analyzed in this study.
